# Supplementary material for: Large mammal population trends in Comoé National Park (1958–2022): Towards understanding their asymmetric decline and recovery in West Africa’s largest savanna park
Source: PLoS One. 2025 May 28;20(5):e0320455. doi: 10.1371/journal.pone.0320455 (PMC12118930; doi:10.1371/journal.pone.0320455)
Supplement: S1 Alternative Language Abstract — (DOCX) [file pone.0320455.s005.docx]

**Large mammal population trends in Comoé National Park (1958-2022): towards understanding their asymmetric decline and recovery in West Africa’s largest savanna park**

**Résumé (Français)**

Le déclin de la faune sauvage en Afrique suscite beaucoup d’intérêt, mais les raisons de ce déclin restent encore difficiles à décrypter. À l’aide de modèles statistiques (GAM) développés sur 25 comptages terrestres et aériens, nous présentons les premiers résultats des tendances temporelles à long terme de populations de grands herbivores d’Afrique de l’Ouest ainsi que des potentiels facteurs expliquant ces variations. Après la création du parc national de la Comoé en 1968, les populations de grands herbivores ont vite augmenté mais ont ensuite décliné, en partie à cause de la crise politique (2002-2011) lorsque la gestion active du parc a cessé. Entre 2010 et 2022, les populations d’hippotragues, de bubales et de cobes Defassa ont quasiment retrouvé les effectifs d’avant la crise. Les populations de cobes de Buffon, d’hippopotames et d’éléphants de savane, autrefois dominantes, sont cependant restées à des niveaux très bas, 10% des effectifs observés entre 1970 et 1980. Les plaines herbeuses ont chuté de 15 à 2% entre 1979 et 2020, altérant probablement les populations convoitant ce type d’habitat comme les cobes de Buffon. Depuis 1962, la population humaine autour du parc et le bétail à l’intérieur ont plus que sextuplé, alors que le nombre de gardes du parc n'a que doublé. Ces facteurs semblent avoir bouleversé l’assemblage des espèces d’herbivores étudiés du parc. Les espèces typiques des savanes arbustives comme les bubales et hippotragues ont maintenant retrouvé les niveaux d’avant la crise, contrairement aux cobes de Buffon et hippopotames, restant à des niveaux très bas probablement en raison de la réduction des plaines herbeuses et de leur caractère grégaire les rendant vulnérables au braconnage. Notre étude plaide pour un accroissement des efforts vers une meilleure compréhension des changements d’habitats et de pression de braconnage opérant dans le parc, avant de réintroduire des espèces disparues comme le lion. Cette étude met en évidence l’importance ainsi que les défis de d’étudier les tendances des populations de grandes herbivores dans des aires protégées et des facteurs de changement qui y sont associés.
