# Supplementary material for: Large mammal population trends in Comoé National Park (1958–2022): Towards understanding their asymmetric decline and recovery in West Africa’s largest savanna park
Source: PLoS One. 2025 May 28;20(5):e0320455. doi: 10.1371/journal.pone.0320455 (PMC12118930; doi:10.1371/journal.pone.0320455)
Supplement: S1 Appendix — (DOCX) [file pone.0320455.s001.docx]

**Supporting information**

**S1 Appendix. Local large mammal extinctions and status of large carnivores**

Black rhinoceros was supposedly reported from the Bouna area in 1905 (Fischer et al. 2002), yet ‘attributed to a huge warthog by Raphael Matta’ (Guillaume, 1959). Rookmaker (2004) argued that the historic range of black rhino was East of present Benin, clearly excluding Comoé NP. Western giant eland was reported as extinct by 1950 (OIPR 2014), who referred to ‘Matta’s observations’, without further details or reference, nor mentioned by Guillaume (1959). Fischer & Linsenmair (2002) stated that there are no records from Comoé. Western giant eland has been reported occurring historically from Senegal to Togo (Planton & Michaux, 2013). Doubts have been also voiced regarding the occurrence of Bongo (*Tragelaphus euryceros*), as mainly reported from outside the present park boundaries, i.e. North of Dabakala (Fischer et al., 2002; Geerling, 1968). In 2017 one individual has been observed with a camera trap inside the park (pers. comm. Lapuente 2017).

Although large carnivores have been reported as prominent since the start of wildlife conservation, only recently efforts have started to assess remaining large carnivore populations (Lapuente 2019; Aglissi et al. 2024). Confusion on their historic presence has continued, we therefore summarize our understanding of literature and observations. Cheetah (*Acinonys jubatus*) was mentioned extinct by 1960 (Fischer et al., 2002), not considered by earlier authors (Geerling, 1968; Lartiges & Poilecot, 1997; Lauginie,1977), whereas Brugière et al. (2016), concluded that cheetah historically never occurred in Comoé. Wild dog (*Canis pictus*) was reported as extremely rare by Lartiges & Poilecot (1997), considered extinct by Brugière et al. (2016), although its continued passage was not excluded by Lapuente (2019), but not observed by Aglissi et al. (2024). The last lion observations have been confirmed from 2010 (Aglissi et al. 2023a), although rumors on its existence have continued to surface, as recently as 2018 (OIPR 2015a; Lapuente 2019). Lion abundance has never been systematically surveyed, with guesstimates of 100 in 1958 (Guillaume, 1959), 100 in 1968 (Geerling 1968) and again 100 in 1987 (Poilecot 1989), surprisingly constant given the three-fold difference in wild herbivore biomass (i.e. potential prey) between those years (Fig 4). Leopard has been widely distributed, estimated as ‘limited number’ in the first years of conservation (Geerling 1968) with five different individuals observed in an area in the SW (Lapuente 2019), and 11 independent captures by Aglissi et al. (2024). Spotted Hyena, estimated at 100 in 1968 (Geerling 1968) is presently the most common large carnivore, and although no population estimates have been made, an estimate of 100 seems has been suggested by Lapuente (2019), see also Aglissi et al. (2024).

**Additional references**

Aglissi, J., Sogbohossou, E.A., Soro, F., Ouattara, S., Sinsin, B. and Bauer, H., 2024. Ecological determinants of spotted hyena Crocuta crocuta occupancy in Comoé National Park, Côte d’Ivoire. *European Journal of Wildlife Research*, *70*(2), p.21.

Lapuente, J. (2019). *Grands Carnivores au Parc National de la Comoé*. Rapport des résultats de la première phase, 2019. OIPR, Côte d’Ivoire.

Lartiges A.**,**P. Poilecot **(**1997). Evolution récente de la grande faune dans le Parc national de la Comoé, en Côte d'Ivoire. *Bulletin Mensuel de l'Office National de la Chasse* (226) : pp. 20-31.

Planton, H. & I. Michaux (2013). Tragelaphus derbianus. Giant Eland. Pp 186-190. in: Kingdon, J. & Hoffmann, M. (eds). *The Mammals of Africa. Vol. VI. Pigs, Hippopotamuses, Chevrotain, Giraffes, Deer and Bovids.*  Bloomsbury Publishing, London.

Rookmaaker, L.C. (2004). Historical distribution of the black rhinoceros (*Diceros bicornis*) in West Africa. African Zoology *39*(1):63–70.
