# Supplementary material for: Large mammal population trends in Comoé National Park (1958–2022): Towards understanding their asymmetric decline and recovery in West Africa’s largest savanna park
Source: PLoS One. 2025 May 28;20(5):e0320455. doi: 10.1371/journal.pone.0320455 (PMC12118930; doi:10.1371/journal.pone.0320455)
Supplement: S1 Table — (DOCX) [file pone.0320455.s003.docx]

**S1 Table. Years of major events in Comoé NP** Sources: Expanded from OIPR (2015a) based on Guillaume (1959), Geerling & Bokdam (1973) and pers. obs.

| **Year** | **Status & Boundaries^[[1]](#footnote-1)^** | **Wildlife / Research** | **Management / Tourism** | **Wider events** |
| --- | --- | --- | --- | --- |
| 1926 | Creation of *Parc de la région Nord* |  |  |  |
| 1942 | *Reserve totale de faune et forêt classée de Bouna* (E.of Comoé R.) and *forêt classée de Kong* (W. of Comoé R.) |  | Trophy hunting remained possible, criticized by some (Guillaume 1959) |  |
| 1950 |  | last Giant Eland seemingly observed |  |  |
| 1953 | Further enlargement of reserve |  | Removal of villages inside the reserve |  |
| 1959 |  | Account Matte/park (Guillaume 1959) | Murder of Raphael Matte, park manager |  |
| 1960 |  |  |  | Independance of Côte d’Ivoire |
| 1963 |  |  | Last village, Biguilaye, removed |  |
| 1968 | Combined *Reserve de Bouna* and *Forêt classée* into a national park of c.1.150.000 ha | First quantitative large herbivore surveys (Geerling & Bokdam 1973) | End of trophy hunting |  |
|  |  |  | Operning of Kafalo safari lodge (NW) |  |
| 1977 | Reduction with 850 ha to 1149150 |  |  |  |
| 1982 | Listing as UNESCO Biosphere reserve |  |  |  |
| 1983 | Listing as UNESCO World Heritage site |  |  |  |
| 1988 |  | Discovery of Chimpanzee in SW forest galleries |  |  |
| 1989 |  | Start Comoé research station |  |  |
| 1992 |  |  | Ganse lodge (SW) ceased operations |  |
| 2000 |  | Creation of permanent research station [*http://comoe-station.org/*](http://comoe-station.org/) |  |  |
| 2002 |  |  | Creation of OIPR parastal park service | Begin of civil war |
| 2003 | Listing as World Heritage site in danger |  |  |  |
| 2010 |  | last lion observed (Aglissi et al. 2024) |  |  |
| 2011 |  |  |  | End of civil war |
| 2014 |  |  | Opening of funding line through the *Fondation Parcs et Réserves de Côte d'Ivoire* |  |
| 2015 |  |  | Start of German development support |  |
| 2016 | Removal from the list of World Heritage sites in danger |  |  |  |
| 2018 | Reduction to 1148 756 km^2^ (West of Comoe R.) |  |  | Area listed as red zone |
| 2019 |  | Start of systematic large carnivore studies by camera traps (Lapuente 2019, Aglissi et al. 2024) | Closure research station  Kafalo safari lodge ceased operations | Jihadist attacks on Kafalo military post (NW corner). |
| 2021 |  |  | Exploratory contacts between the Gov.of Côte d’Ivoire with African Parks for delegated management |  |

1. Note that governance has remained public throughout this period [↑](#footnote-ref-1)
