# Supplementary material for: Large mammal population trends in Comoé National Park (1958–2022): Towards understanding their asymmetric decline and recovery in West Africa’s largest savanna park
Source: PLoS One. 2025 May 28;20(5):e0320455. doi: 10.1371/journal.pone.0320455 (PMC12118930; doi:10.1371/journal.pone.0320455)
Supplement: S2 Table — (DOCX) [file pone.0320455.s004.docx]

**S2 Table. Data description of large herbivore counts in Comoé NP (1958-2022)**

| **Year** | **Counting**  **Period** | **Species**  **targeted in count** | **Total,**  **Transect, Indices^1^**  **(sampling %)** | **Aerial ^2^/ Terrestrial**  **Transport means** | **Area covered** | **Notes and interpretations** | **Author** | **Reference (cited)**  **(if different from author)** |
| --- | --- | --- | --- | --- | --- | --- | --- | --- |
| 1958 | 1954-58 | all, excl. bushbuck, warthog, | guesstimate | T | Reserve de Bouna (East of Comoé river, 950 000 ha) | Reported exchange with park supervisor | Raphael Matta pers. comm (1958) | Guillaume (1959) |
| 1968 | Jan - May | kob  all | TR (kob), guesstimate (others) | T car | ≤1 km from Comoé river totaling 400 km^2^ | Extrapolations from Comoé valley | Geerling (1968) | Geerling & Bokdam (1973) |
| 1974 |  | all | TO | T |  | Guesstimates | Lauginie (1975) | Lauginie (2007) |
| 1977-81 |  | all | TR (5-6%) | A |  | kob has been ‘reinterpreted’ at 50 000 | FGU Kronberg (1979) | Steinhauer-Burkart (1987)  Muehlenberg & Roth (1985) |
| 1977-81 |  | all | TR (xx) | T foot |  |  | FGU Kronberg (1979) | Steinhauer-Burkart (1987) |
| 1977-81 |  | all |  | T car |  |  |  |  |
| 1978 | ? | hippo | TO | T boat | 185 km river |  |  | Roth et al. (2004) |
| 1981 | ? | hippo | TO | A | 1 x 225 km river |  |  | Roth et al. 2004) |
| 1984 | ? | hippo | TO | A | 1 x 225 km river |  |  | Roth et al. (2004) |
| 1984 | March | all | TR (3.3%) | A |  |  | Poilecot (1989) | Fischer & Linsenmair (2001) |
| *1987* | *Feb-June* | *all* | *TR (0.9%)* | *T car* | *645 km of tracks, 160 m band: 103 km^2^ covered* |  | Poilecot (1989), Poilecot et al. (1991), Lauginie et al. (1995) |  |
| 1988  1989 | Jul, Aug, Nov  Feb, Mar, May-June | all  all | TR (6.5%)  TR (6.5 %) | A  A |  |  | Lauginie et al. (1995)  Lauginie et al. (1995) | Lauginie (2007) |
| 1995 |  | all | TR (xx) | T |  |  | Lartiges et Poilecot (1997) / Lauginie et al. (1995) | Lauginie (2007) |
| 1997-98 | Feb-May  Mar-May | all | TR (1%, 2.25% kob and waterbuck) | T (car) |  |  | Fischer & Linsenmair (2001) |  |
| *2002* | *April* | *hippo* | *hippo* | *T* | *Survey over 34km (S.Comoe),* | *‘47 hippo counted, suggesting decline’* | Roth et al. (2004) |  |
| 2010 | March | all | TR (6.21%) | A |  |  | WCF (2010) |  |
| *2012* | *March -August* | *all* | *TR (<1*%*)* | *T foot* | *296 km of transects* |  | WCF (2012) |  |
| 2014 | April | all | TR (6.21%) | A |  |  | Kouakou et al. (2014) |  |
| 2016 | March | all | TR (20%) | A |  |  | Bouché (2016) |  |
| 2016 | March | hippo | TO | A | 1 x 225 km river |  | Bouché (2016) |  |
| 2016 | Mar-Apr  Jul - Aug | elephant | IN | T foot | Forest patches in SW(1036 km^2^), resp. 144-256 km transect | dung count | OIPR (2017a) |  |
| 2017 | March | elephant | IN | T foot | Forest patches in SW (1036 km^2^), 230 km transect | dung count | OIPR (2017b) |  |
| 2018 | March | elephant | IN | T foot |  | dung count | OIPR (2018) |  |
| *2018-19* | *May-June*  *Feb - Mar* | *African buffalo* | *IN* | *T* |  | *droppings were used as base* | Atta et al. (2020) |  |
| 2019 | April May | all | TR (20%) | A |  |  | OIPR (2020) |  |
| 2019 | May | hippo | TO | A | 230, resp. 225 km river N-S, S-N | highest number of the two trajectories | OIPR (2020) |  |
| 2021 | May-June | elephant | IN | T foot | Forest patches in SW (1036 km^2^), 116 km transect | dung count | OIPR (2021) |  |
| 2022 | Mar-April | all (-hippo) | TR (15%) | A |  |  | Sabdano et al. (2022) | Note major differences in numbers of elephant presented by Delplanque and Sabdano for the aerial count, other species only minor differences. |
|  |  | *hartebeest, buffalo, kob, waterbuck, elephant, roan, warthog* | *obligue camera count (OCC)* | *A* |  | *148239 images processed with deep learning. OCC numbers vs count:*  *kob (+241%), warthog (+163%)*  *buffalo (+17%)*  *waterbuck (-2%)*  *hartebeest (-7%).* | *Delplanque et al. 2024* |  |
| *2022* | *Feb-June* | *Leopard, spotted hyena* | *1 camera per 25 km square* | *T camera trap* | *3125 km^2^ (125 5*5 km grid) centre-south* |  | *Aglissi et al. 2024* |  |

Notes: 1. TO: Total count; TR: Transect count; IN: indices (indirect observations, droppings mainly);

1. A: Aerial; T: Terrestrial; Aerial by (high-wing Cessna 4-6 seat) airplane
2. In italics *(1984, 2002, 2012, 2018-19, 2022 (oblique camera count), 2023)*, not included in analysis and Figures 2,3, because of cover < 1% sampling, non-random sampling, incomplete cover or parallel interpretations. See also text, methods.

**Additional references**

Bouché, P. (2016). Comptage aérien de la faune du Parc National de la Comoé et des deux zones de biodiversité. Unpublished report, OIPR, GIZ, Abidjan, Cote d’Ivoire

Kouakou Y. C., Maho N. R. Tiédoué R., Ouattara, A., Vergnes V. et Normand E. (2014). Etat de conservation du Parc National de la Comoé et de sa zone périphérique : Rapport de l’inventaire faunique par survol du 17 au 24 Avril 2014. Rapport WCF, Abidjan.

Lauginie, F. (1975). Composantes du milieu naturel et environnement socio-économique du Parc national de la Comoé, en Côte d'Ivoire. Propositions de schéma d’aménagement. SEPN/BD :PA, Abidjan, 97 pp.

Muehlenberg, M. & H.H. Roth. (1985). Comparative investigations into the ecology of the kob antelope *Kobus kob kob* (Erxleben 1777) in the Comoe National Park, Ivory Coast. *S.Afr.Wild.Res.* 15 : 25-31.

OIPR (2017a). Distribution et Estimation de la Population d’Eléphant dans les Ilots Forestiers et Galeries Forestières de la moitié Sud Du Parc National De La Comoe et dans sa Périphérique : Etude pilote. Unpublished report, Direction Nord-Est, OIPR, Cote d’Ivoire. Cote d’Ivoire.

Roth, H.H, [B. Hoppe-Dominik](https://www.tandfonline.com/author/Hoppe-Dominik%2C+Bernd), [M.Mühlenberg](https://www.tandfonline.com/author/M%C3%BChlenberg%2C+Michael), [B.Steinhauer-Burkart](https://www.tandfonline.com/author/Steinhauer-Burkart%2C+Bernd) & [F.Fischer](https://www.tandfonline.com/author/Fischer%2C+Frauke) (2004). Distribution and status of the hippopotamids in the Ivory Coast. *African Zoology* 39: 211-224.

Sabdano, N., J. Linchant, L.Eisendrath & X. Vincke. (2022). Inventaire aérien du Parc national de la Comoe et des sites de biodiversité de Waringue et de Mont Tingui. OIPR, KfW, Abidjan, Côte d’Ivoire.

WCF (2010). Etat des ressources naturelles du Parc National de la Comoé et de sa zone périphérique. Rapport de l’inventaire faunique par survol aérien mars 2010. WCF OIPR GIZ.

WCF (2012). Suivi Ecologique au Parc National de la Comoé. Rapport de l’inventaire pédestre de la faune (mars – aout 2012). WCF OIPR.
