# Supplementary material for: Large mammal population trends in Comoé National Park (1958–2022): Towards understanding their asymmetric decline and recovery in West Africa’s largest savanna park
Source: PLoS One. 2025 May 28;20(5):e0320455. doi: 10.1371/journal.pone.0320455 (PMC12118930; doi:10.1371/journal.pone.0320455)
Supplement: Attachment — Submitted filename: Comoe-Response to reviewers.docx [file pone.0320455.s006.docx]

**Response to reviewers’ comments**

***Below we react (text in italics) on each of the points made by the two reviewers and editor. In the track-changes version you will find the detailed text changes. In the feed-back we also mentioned the additional references have also been mentioned in the feed-back.***

**Additional Editor Comments:**

Your manuscript represents a tremendous endeavor to highlight population trends and drivers in a part of the world that has been challenging to study. The reviewers and I agree that your manuscript should make a valuable contribution to the literature but would benefit from a few considerations and revisions. Below you will find reviews from two reviewers with expertise in large mammal conservation in savanna Africa and one with particular expertise in statistics and data analysis. Both reviewers provide helpful suggestions for the context of your findings, as well as some analytical suggestions from Reviewer 2.

*Thank you very much for this constructive feed-back*

**5. Review Comments to the Author**

Reviewer #1: The manuscript describes interesting wildlife recovery patterns in a protected area of extreme regional importance. While the datasets used by the authors to describe and interrogate the observed wildlife trends are of very variable nature and quality, as the authors openly acknowledge, the authors appear to have done their best to accommodate the obvious flaws and variability in those datasets, by using GAMs and by interpreting the results with the required caveats.
I would therefore generally recommend to accept this manuscript for publication.

1. However, I would strongly recommend to further strengthen the discussion by relating the observed wildlife recovery trends for the various species to common ecological concepts described for African savanna herbivore communities, which could help to explain the observed patterns.

*Thank you, we have followed this useful advice, see the specific responses below. Especially the section* ***Understanding large herbivore population trends*** *has been developed and further divided into*

*General conservation efforts, Selective predation, Selective poaching, and Vegetation – large mammal relationships. Based on the suggestions of the reviewer we also expanded the earlier section of ‘lion reintroduction’ into: ‘****Rewilding initiatives and potential of lion reintroduction’****.*

2. The possible relationship between low elephant numbers and a decrease in open habitats is mentioned in once sentence (lines 374-376), however, no references are given and the effect this could have on other herbivore species is not discussed. There is strong evidence for the importance of such interactions, however (e.g. de Boer et al, 2015), and this could be discussed in more detail.

*We realize we have not been sufficiently explicit about elephant – vegetation relations. We therefore added the proposed reference (de Boer et al. 2015, S.Africa) in the sentence that we elaborated, also adding the reference of Daskinet al. 2016 from Gorongosa (Mozambique) whereas we kept the reference of Dublin et al. 1990 (from Kenya-Tanzania).*

3. The situation of hippopotamus in the park is discussed in some detail, but the authors do not evaluate the ecological role of the species, and any effects their population collapse may have on other species. Like elephants, common hippo are considered to be an ecosystem engineer (e.g. Voysey et al, 2023), and their facilitative role for smaller herbivores has also been documented for Western kob (e.g. Verweij et al, 2006). This could be discussed in more detail.

*Thank you for the suggestion to expand the discussion and reference to Verweij et al. 2006. We added the reference to Voysey et al. 2023, and shortly described possible ways to restore grazing lawns, based on recent experiences from South Africa (Schroder et al. 2024). This is now also mentioned in the Conclusion and recommendations section.*

4. Furthermore, it has been well-documented in neighbouring Ghana that the local extirpation of lions (and leopards) led to dramatic trophic cascades, with meso-predators like baboons increasing their numbers 4-5-fold, which in turn led to a dramatic decline in numbers of medium-sized ungulates (Brashares et al, 2010). The local extinction of lions is mentioned several times by the authors, but no possible link to trophic cascades is explored. Baboon predation of herbivores particularly targets fawns of species like kob that hide fawns in tall grasses at the edge of grazing lawns, conversely to larger plains herbivores like hartebeest, or also the roan, for which even newborns follow herds within hours and are not overly susceptible to baboon predation. This potential role of meso-predators, which may have become hyperabundant following the local extirpation of top predators such as lions, is not discussed at all.

*We have elaborated a paragraph on possible predation on mesoherbivores, as reason for the timid recovery of kob (in the section* ***Understanding large herbivore population trends).*** *Here we extensively refer to the study of Brashares et al. (2010) from neighboring Ghana where baboon numbers exploded impacting numbers of mesoherbivores. We included in S1 Fig a new graph with a GAM analysis of the numbers of baboon with time in Comoé park (albeit grossly underestimated by the later aerial surveys) and concluded that although lion is locally extirpated, leopard (and spotted hyena) are not (Agkissi et al. 2024) and have likely kept baboon numbers low.*

5. On the other hand, and also regarding lions, the links the authors are trying to establish between low kob numbers and potential lion reintroduction plans seem construed. Kobs are neither within the preferred prey range of lions, which is in the range of 190–550 kg (Hayward & Kerley (2005), nor was kob to be found an important prey species in dietary analyses across West & Central Africa (Bauer et al, 2008). As can be seen in Bauer et al (2008), larger-bodied species such as hartebeest and roan are generally much more important.

*We further developed the reasoning by using the references the reviewer proposed, as well as others, most notably the study from Comoe of Bodendorfer et al. (2006), and those from other open wooded savana areas with dominance of kob (Faro and Waza in Cameroon, Manovo-Gounda-St Floris in CAR), see Wanzie (1987), Ruggiero 1991, Breuer 2005. We also refer to more specialized studies conducted on lion in Comoé park (Aglissi et al. 2024).*

6. Lastly, it would also be beneficial to the readers if the authors discussed their findings in the context of recovery trends and patterns from other African protected areas. Some really interesting works on this matter come from Gorongosa NP in Mozambique, for example, where contemporary herbivore community structure and biomass also differs radically from the previous documented baselines (e.g. Pansu et al, 2019). There are other, similar examples from different systems, and the manuscript would really benefit from a discussion showing that such skewed recovery favouring only certain species appears to be a phenomenon which is more common than may have been anticipated, and may require specific research and management interventions to restore pre-depletion faunal assemblages.

*Thank you for recommending the comparison with Gorongosa NP (Mozambique) as well as other protected areas with documented large mammal collapses and subsequent recoveries. We introduced a section ‘Rewilding initiatives and potential of lion reintroduction’. We delved into the literature of its recovery in Gorongosa (including Stahlmans et al. 2019 in this journal), as well as other rewilding examples (Zakouma, Waza, …). We also used examples of Gorongosa (Daskin et al. 2016) in other sections of our manuscript (Vegetation – large mammal relations).*

7. Specific literature to consider in the discussion:
   Bauer, H., Vanherle, N., Di Silvestre, I., & De Iongh, H. H. (2008). Lion–prey relations in West and Central Africa. Mammalian Biology, 73(1), 70-73.

*integrated*
Brashares, J. S., Prugh, L. R., Stoner, C. J., & Epps, C. W. (2010). Ecological and conservation implications of mesopredator release. Trophic cascades: predators, prey, and the changing dynamics of nature, 221-240.

*integrated*
de Boer, W. F., Van Oort, J. W., Grover, M., & Peel, M. J. (2015). Elephant-mediated habitat modifications and changes in herbivore species assemblages in Sabi Sand, South Africa. European Journal of Wildlife Research, 61, 491-503.

*integrated*
Fritz, H., Duncan, P., Gordon, I. J., & Illius, A. W. (2002). Megaherbivores influence trophic guilds structure in African ungulate communities. Oecologia, 131, 620-625.

*Not integrated, but see Owen-Smith 1988 , more explicit re. (non-) predation on megaherbivores, below*
Hayward, M. W., & Kerley, G. I. (2005). Prey preferences of the lion (Panthera leo). Journal of zoology, 267(3), 309-322.

*integrated*
Pansu, J., Guyton, J. A., Potter, A. B., Atkins, J. L., Daskin, J. H., Wursten, B., ... & Pringle, R. M. (2019). Trophic ecology of large herbivores in a reassembling African ecosystem. Journal of Ecology, 107(3), 1355-1376.

*Not integrated, instead Stalmans et al. 2019, more explicit about the survey history and results in Gorongosa, rendering comparison with Comoe NP easier*
Verweij, R., Verrelst, J., Loth, P. E., MA Heitkönig, I., & MH Brunsting, A. (2006). Grazing lawns contribute to the subsistence of mesoherbivores on dystrophic savannas. Oikos, 114(1), 108-116.

*maintained*

Voysey, M. D., de Bruyn, P. N., & Davies, A. B. (2023). Are hippos Africa's most influential megaherbivore? A review of ecosystem engineering by the semi‐aquatic common hippopotamus. Biological Reviews, 98(5), 1509-1529.

*integrated*

*Thank you very much for this comprehensive list with useful references. Below you find the other references that further to the recommendations we used.*

Additional references:

Bodendorfer T, Hoppe-Dominik B, Fischer F, Eduard Linsenmair K. Prey of the leopard (Panthera pardus) and the lion (Panthera leo) in the Comoé and Marahoué National Parks, Côte d'Ivoire, West Africa/ Mammalia. 2006 Mar;70(3-4):231-46.

Breuer T. Diet choice of large carnivores in northern Cameroon. African journal of ecology. 2005 Sep;43(3):181-90.

Daskin JH, Stalmans M, Pringle RM. Ecological legacies of civil war: 35‐year increase in savanna tree cover following wholesale large‐mammal declines. Journal of Ecology. 2016 Jan;104(1):79-89.

Delplanque, A., Linchant, J., Vincke, X., Lamprey, R., Théau, J., Vermeulen, C., Foucher, S., Ouattara, A., Kouadio, R. and Lejeune, P., 2024. Will artificial intelligence revolutionize aerial surveys? A first large-scale semi-automated survey of African wildlife using oblique imagery and deep learning. *Ecological Informatics*, p.102679.

Fraticelli C, Zayed AA, Leirs H, Bauer H. Lions select larger prey in a Central African protected area with increasingly effective management. Ecology and Evolution. 2024 Jul;14(7):e70062.

Moritz M, Hunter CE, Scholte P. Reflooding the coupled human and natural system of the Waza-Logone Floodplain, Cameroon. Frontiers in Conservation Science. 2024 Sep 24;5:1384747.

Owen-Smith RN. Megaherbivores: the influence of very large body size on ecology. Cambridge university press; 1988.

Ruggiero RG. Prey selection of the lion (Panthera leo L.) in the Manovo-Gounda-St. Floris National Park, Central African Republic. Mammalia. 1991;55(1):23-33.

Scholte P, Adam S, Serge BK. Population trends of antelopes in Waza National Park (Cameroon) from 1960 to 2001: the interacting effects of rainfall, flooding and human interventions. African journal of ecology. 2007 Sep 1;45(3).

Schroder B, Schroder NA, Van Langevelde F, Prins HH. Effects of mineral addition on the establishment of grazing lawns in a nutrient poor savanna. bioRxiv. 2024:2024-01

Stalmans ME, Massad TJ, Peel MJ, Tarnita CE, Pringle RM. War-induced collapse and asymmetric recovery of large-mammal populations in Gorongosa National Park, Mozambique. PloS one. 2019 Mar 13;14(3):e0212864.

WANZIE, Chris. Mortality factors of Buffon’s kob Kobus kob kob (Erxleben) in Waza National Park, Cameroon. Mammalia 1986; 50(3): 351-356.

**Reviewer #2**: Trends in large herbivore populations in Comoé National Park were investigated in relation to various drivers through a data set spanning more than six decades. Large herbivore populations are claimed to have shown a pattern of asymmetric recovery and decline based on environmental changes, poaching and management strategies during periods of political instability. The study further claims that different species are differently affected by the described drivers with some species exhibiting higher levels of recovery than others.

Against the backdrop of ecological management in West Africa, where long-term data are often found to be scarce, these claims are significant for conservational efforts and provides a rare insight into the relationship and between human activity, habitat changes, and wildlife trends which could potentially lead to changes and developments in the spheres of conservational policies.

The study is well placed within the context of existing literature, acknowledging the comparable studies from Central and East Africa, as well as similar studies investigating drivers of wildlife decline. Referencing older works are expected in a long-term wildlife study and relevant newer studies were introduced to show the use of more recent statistical techniques. One technique that could be considered to enhance the discussion, would be Bayesian approaches.

Overall, the authors have managed to effectively describe the knowledge gap that their research addresses.
The data analyses were found to largely support the claims of this manuscript and generalized additive models (GAMs) are appropriately applied in this non-linear trend environment for heterogeneous data.
Data is obtained from various sources and the authors have done well to acknowledge inconsistent methodologies and justifications for the inclusion of such data. The rarity of long-term data is an acceptable rationale, and this study seems to be the first of its kind for this region, potentially providing important and necessary insights into population trends. For future studies, one would hope to see greater effort to harmonize data for improved reliability.
The methodology is sufficiently described for reproduction, and the manuscript is generally well-organized. The manuscript is a valuable contribution to conservation science and given the suggested adjustments/addressing the highlighted concerns below, the study has merit for eventual publication.

*Thank you very much for this positive feed-back. As suggested, we have now mentioned in the Discussion section that i) Bayesian models may also be used to analyze long-term wildlife trends, ii) more effort should be paid in data standardization to improve our ability to examine drivers of wildlife trends.*

We are of the opinion that the following aspects warrant attention:
1. The heterogeneity of the data, while acknowledged, introduces uncertainty in the results, especially where less consistently surveyed species are concerned. Inconsistent sampling techniques may limit the comparability of population estimates over time.

*Further to the reviewer’s comment we included in S2 Table, and referred to in the main text (both methods and discussion), the recently published study of Delplanque et al. 2024 that presents for the 2022 aerial count (already included) a parallel oblique camera count. This first ‘parallel count’ allows further appreciation of possible uncertainties in the presented results, in particular of the smaller large herbivores kob and warthog. Numbers of larger herbivores were remarkably similar We also would like to refer to the discussion on the parallel counting methods (aerial versus terrestrial) of savanna elephant (lines 283-285), as well as of African buffalo (aerial count – indirect dung count) (lines 349-351).*

2. Potential interaction effects of combining multiple drivers should be investigated for deeper insights. The authors note that models incorporating all drivers are not suitable, the importance of individual variables might be overestimated.

*As we mentioned in the methods section, we were constraint by the existing data on potential drivers. To run models with an acceptable degree of freedom, we had to accept, although it was frustrating, to run separate regressions to test for the effects all (potential) drivers, including those (political changes, poaching) which may have led to abrupt changes. That being said, we agree with the referee’s comment that such a procedure might lead to overestimate of some drivers. This is now mentioned in the Discussion.*

3. Cubic B-spline covariance structure and standard assumptions of GAMs might require further investigation for data on ecological trends where instances of political crises could potentially lead to abrupt changes.
*We agree, and have taken this further up, see the description in the last paragraph of the data analysis section that we changed into:*

*We performed diagnostics for all fitted generalized additive models (GAM) on large mammal populations estimates and drivers. We used the ‘k.check’ and ‘gam.check’ functions to i) control that our basis dimension choices for the smooth effects were adequate, ii) get information on the convergence of the smoothness selection optimization, and iii) run diagnostic tests on the distribution of residuals and the variance of residuals against linear predictors [27]. For all other models (i.e., GLS, GLM and linear regression), we checked that residuals fulfilled statistical requirements including the distribution of residuals against fitted values and the lack of temporal autocorrelation using the ‘acf’ functions [28]. All analyses were performed using R 4.3.1 [29].*

*We also elaborated the discussion on the impact of the 2002-2011 political crisis in Côte d’Ivoire, reasoning that the lack of surveys between 1998 – 2010 prevented a more detailed assessment. Based on various observations on ceasing management already since the late 1980s (Fischer & Linsenmaier 2001), we suggest that the impact was more a decades’ long continuum than a ‘shock’.*

4. Confidence intervals are broad and should be interpreted with caution.
*We fully agree, as we mentioned in Results (lines 265, 266), and Discussion (355), we emphasized the broad confidence intervals. We further emphasized this by adding this remark in the Conclusions and recommendations (line 537).*

5. Graphical displays, such as Figure 2 B is unclear. Although it is acknowledged that the authors might have been aiming to keep the y-axis indices the same for both graphs A and B, we believe that it might be more sensible to shrink the indices of the y-axis of graph B to a more sensible ceiling of 2000.

*We agree that the quality of the figures in the submission was not sufficient, and provided high-quality version this time. We would like to keep the y-axis indices the same, emphasizing the importance of the values of total biomass (livestock << wild). If considered decisive, we can change this however.*

6. Aligned with the requirement of the journal, we would have liked to a see a tabular summary such as S3 Table incorporated into the manuscript itself and not merely included in the appendix.

*Further to this observation, we included S3 Table in the main text (as Table 1).*

7. As a point of interest, one wonders what the effect of temperature could be on population trends. It is noted that average temperature is between 28 and 34 degrees, but would this variable not also be a driver against the backdrop of climate change?

*In the section* ***Challenges in analyzing potential drivers*** *we extended the discussion of average rainfall as potential driver, by mentioning the potential importance of temperature and the challenged to obtain (historical) data.*

8. Although a detailed account of the sources and methods used for data analyses is supplied, depositing the raw data in an accessible repository would enhance transparency. Furthermore, simplifying key findings with summary tables could enhance readability.

*We understand the referee’s reasoning and expect that the S2 Table provided as a supplementary material gives a comprehensive view for readers of all sources we gathered and used to create the data set. 16 out of 27 sources mentioned in the S2 Table came from unpublished reports for which we do not hold copyrights. We therefore propose to readers who are they are interested in getting them to contact the corresponding author. We have added a note at the end of the paper: Upon reasonable request to the corresponding author, the here used data will be shared. (Please advice where this sentence can be inserted, we have put in tentatively at the end of the Acknowledgements).*

FIGURE 1

2. We note that Figure 1 in your submission contain map images which may be copyrighted. All PLOS content is published under the Creative Commons Attribution License (CC BY 4.0), which means that the manuscript, images, and Supporting Information files will be freely available online, and any third party is permitted to access, download, copy, distribute, and use these materials in any way, even commercially, with proper attribution. For these reasons, we cannot publish previously copyrighted maps or satellite images created using proprietary data, such as Google software (Google Maps, Street View, and Earth). For more information, see our copyright guidelines: [http://journals.plos.org/plosone/s/licenses-and-copyright](https://eur01.safelinks.protection.outlook.com/?url=http%3A%2F%2Fjournals.plos.org%2Fplosone%2Fs%2Flicenses-and-copyright&data=05%7C02%7Cpaul.scholte%40giz.de%7Cac098afa4d604a7c514108dd199b412f%7C5bbab28cdef3460488225e707da8dba8%7C0%7C0%7C638694876804871782%7CUnknown%7CTWFpbGZsb3d8eyJFbXB0eU1hcGkiOnRydWUsIlYiOiIwLjAuMDAwMCIsIlAiOiJXaW4zMiIsIkFOIjoiTWFpbCIsIldUIjoyfQ%3D%3D%7C60000%7C%7C%7C&sdata=EaSc7mBg9mEwT%2FE5Nf5XxO9ny%2Bta1wN0U80G1%2B2C2Ko%3D&reserved=0).
 
*Attached you find the copyright form, filled it by the Comoe national park authorities*

CHANGES IN REFERENCE LIST

3. Please review your reference list to ensure that it is complete and correct. If you have cited papers that have been retracted, please include the rationale for doing so in the manuscript text, or remove these references and replace them with relevant current references. Any changes to the reference list should be mentioned in the rebuttal letter that accompanies your revised manuscript. If you need to cite a retracted article, indicate the article’s retracted status in the References list and also include a citation and full reference for the retraction notice.

*See new list under response to reviewer 1*
